# Birth and Neonatal Transition in the Guinea Pig: Experimental Approaches to Prevent Preterm Birth and Protect the Premature Fetus

**DOI:** 10.3389/fphys.2018.01802

**Published:** 2018-12-11

**Authors:** Jonathan J. Hirst, Hannah K. Palliser, Julia C. Shaw, Gabrielle Crombie, David W. Walker, Tamas Zakar

**Affiliations:** ^1^Mothers and Babies Research Centre, Hunter Medical Research Institute, Newcastle, NSW, Australia; ^2^School of Biomedical Sciences and Pharmacy, University of Newcastle, Newcastle, NSW, Australia; ^3^School of Health and Biomedical Sciences, RMIT University, Bundoora, VIC, Australia; ^4^School of Medicine and Public Health, University of Newcastle, Newcastle, NSW, Australia

**Keywords:** guinea pig, progesterone, preterm birth, IUGR, neurodevelopment, neurosteroids, animal model

## Abstract

The guinea pig (Cavia porcellus) displays many features of gestational physiology that makes it the most translationally relevant rodent species. Progesterone production undergoes a luteal to placental shift as in human pregnancy with levels rising during gestation and with labor and delivery occurring without a precipitous decline in maternal progesterone levels. In contrast to other laboratory rodents, labor in guinea pigs is triggered by a functional progesterone withdrawal, which involves the loss of uterine sensitivity to progesterone like in women. In both species the amnion membrane is a major source of labor-inducing prostaglandins, which promote functional progesterone withdrawal by modifying myometrial progesterone receptor expression. These similar features appear to result from convergent evolution rather than closer evolutionally relationship to primates compared to other rodents. Nevertheless, the similarities in the production, metabolism and actions of progesterone and prostaglandins allow information gained in pregnant guinea pigs to be extended to pregnant women with confidence. This includes exploring the effects of pregnancy complications including growth restriction and the mechanisms by which stressful conditions increase the incidence of preterm labor. The relatively long gestation of the guinea pig and the maturity of the pups at birth particularly in brain development means that a greater proportion of brain development happens *in utero*. This allows adverse intrauterine conditions to make a sustained impact on the developing brain like in compromised human pregnancies. In addition, the brain is exposed to a protective neurosteroid environment *in utero*, which has been suggested to promote development in the guinea pig and the human. Moreover, *in utero* stresses that have been shown to adversely affect long term neurobehavioral outcomes in clinical studies, can be modeled successfully in guinea pigs. Overall, these parallels to the human have led to increasing interest in the guinea pig for translational studies of treatments and therapies that potentially improve outcomes following adverse events in pregnancy and after preterm birth.

## Introduction

Ethical considerations restrict possible experimental interventions for studying human reproductive physiology and pathophysiology at the whole organism level. These considerations are even more limiting when the effects of adverse pregnancy conditions are investigated on fetal development and pregnancy outcomes. Animal models are, therefore, indispensable to obtain information on mechanisms that control fetal and maternal health, organ development and gestational length. Research exploring fetal development benefits particularly from animal models, since pregnancy causes major changes in the cardiovascular, immune and endocrine systems of the mother, which are critical to fetal maturation, pregnancy maintenance and the optimal timing of birth. Furthermore, birth involves precisely coordinated interactions between the mother and fetus leading to structural remodeling and functional alterations of the myometrium, cervix and the fetal membranes, which can be understood in their complexity by observing, instrumenting and experimentally challenging pregnant model animals *in vivo*. Studying gestational changes that lead to term and preterm birth is complicated by marked species differences in placentation, the endocrinology of pregnancy and the maturity of the fetus at birth, which requires careful evaluation of prospective animal models to assess the potential for translation to humans.

**FIGURE 1:**
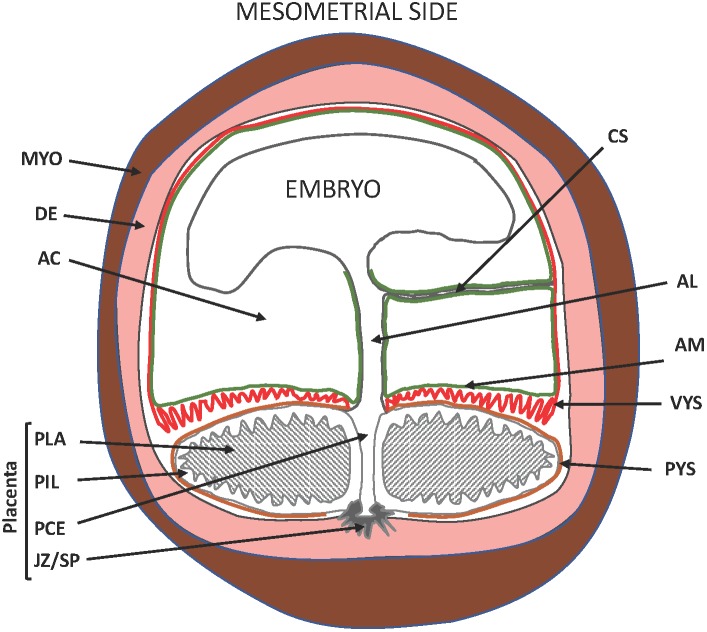
Schematic diagram of the pregnant guinea pig (Neotropical caviomorph) uterus. AC, amniotic cavity; AM, amnion membrane (green), site of prostaglandin production; VYS, visceral yolk sac (red), site of prostaglandin metabolism; PYS, parietal yolk sac (orange); AL, allantois developing to form the umbilical cord; CS, connecting stalk; PLA, placental labyrinth; PIL, placental interlobium; JZ/SP, junctional zone developing to form the sub-placenta; PCE, central excavation of the placenta; DE, decidua/endometrium (pink); MYO, uterine muscle (brown). Blood vessels are not shown. Adapted from Oliveira et al. (2008).

Interest in the guinea pig (Cavia porcellus) for studies of pregnancy and neonatal outcome is growing with the appreciation of the drawbacks associated with rats and mice. Most conspicuously, rats and mice deliver after systemic progesterone withdrawal caused by a sharp drop in maternal progesterone levels as opposed to women, who give birth without a decline of circulating maternal progesterone. Furthermore, rats and mice deliver a very immature fetus compared to women. Guinea pigs give birth without a precipitous decline in maternal progesterone levels at term (Challis et al., 1971; Nielsen et al., 2016). They exhibit luteo-placental shift in progesterone production before mid-pregnancy (Csapo et al., 1981) and fail to respond to exogenous progesterone with prolonged gestation, similarly, to women (Zarrow et al., 1963; Porter, 1970). The guinea pig, therefore, is a rodent species (Sullivan and Swofford, 1997) that delivers its young after functional, and not systemic, progesterone withdrawal like primates. Haemochorial placentation is a further important parallelism between guinea pigs and primates, but differences also exist. These include the presence of the placental interlobium, which is the primary site of hormone production, the subplacenta, which attaches the placenta to the uterine wall, and the visceral yolk sac, which replaces the receding blastocystic wall and the chorion leave (Kaufmann and Davidoff, 1977; Oliveira et al., 2008; Carter and Enders, 2016; Figure 1). Nonetheless, the similarities of progesterone action in humans and guinea pigs have raised the possibility that guinea pigs might serve as experimental models to study parturition with particular relevance to humans.

The guinea pig with its comparatively long gestation and large fetal size is particularly suitable for studies of late gestation fetal development. Importantly, despite the smaller litter size of the guinea pig, typically 2–4, the relatively advanced development of the fetus at birth and the substantial combined weight of litters places considerable burden on the placenta and uterine blood flow (Shaw et al., 2016; Morrison et al., 2018). This creates vulnerability to a reduction in nutrient supply allowing the study of placental insufficiency, which may contribute to the risk of early delivery as in human pregnancy (Morrison et al., 2018). Patterns of brain development in the guinea pig are more similar to the human than to altricious rodents like mice and rats with greater development occurring *in utero* and therefore influenced by the placenta and uterine environment (Dobbing and Sands, 1970; Tolcos et al., 2011). Postnatal development allows for long-term outcome and behavior to be determined in a reasonable timeframe using established and well characterized techniques for the guinea pig offspring (Kapoor and Matthews, 2005).

There are further important similarities between guinea pig and human pregnancy that are not shared by species in other models. As mentioned, the guinea placenta synthesizes progesterone, and production is maintained until term (Challis et al., 1971). The resultant high plasma progesterone concentration contributes neurosteroids and neurosteroid precursors to the fetal brain (Hirst et al., 2014). There is no decline in progesterone production before the onset of labor and, as seen in primate pregnancy, labor onset appears to be triggered by a loss of progesterone sensitivity (Porter, 1970; Haluska et al., 1997). Preparation for labor also involves a marked relaxation and dilation of the pubic symphysis which can be a useful indication that labor is approaching. The drawback of limited DNA sequence information for the guinea pig compared to mice has now largely been overcome with the sequencing of the guinea pig genome (GenBank: AAKN00000000.2, WGS assembly Cavpor3.0). The following sections detail key aspects of guinea pig pregnancy and labor and highlight the value of this species to model preterm labor and maternal-placental-fetal brain interactions. The authors hope that highlighting some key characteristics will further raise interest in the use of guinea pigs for the advancement of perinatal research.

## Guinea Pig Model of Human Labor and Preterm Birth

### Evolutionary Relations of Progesterone Withdrawal in Guinea Pigs and Humans

The translation of findings in animal models is usually based on homology with the corresponding features in humans. Intuitively, close phylogenetic relationship is likely to result in homologies that can be utilized for modeling. Humans and the most widely used laboratory rodents (mice, rats, rabbits, guinea pigs) belong to the same superorder, the Euarchontoglires, which split to the clades Euarchonta (including primates) and Glires (including rodents) about 91 million years ago (Murphy et al., 2007). In all these species, progesterone is pivotally important for establishing and maintaining pregnancy, and withdrawal of progesterone is critical for parturition to occur. The mechanisms for producing, maintaining and withdrawing progesterone diverged, however, in the two clades after evolutionary separation. In many rodents, progesterone is produced by the corpus luteum during the entire pregnancy, and luteolysis at term results in a drop of circulating maternal progesterone level. This causes systemic progesterone withdrawal, which precipitates birth. In primates, the source of progesterone shifts from the ovaries to the placenta in early pregnancy, and high progesterone levels are maintained throughout gestation (Haluska et al., 1997). Importantly, progesterone production by the placenta does not decline prior to birth in Catarrhine primates (Nnamani et al., 2013). Instead the fall in progesterone action is said to be functional involving local mechanisms such as target tissue metabolism and changing progesterone receptor expression and function (Zakar and Mesiano, 2011). In agreement with these processes, progesterone administration can prolong pregnancy beyond term in rodents, but not in humans (see below) or higher non-human primates. Using Laurasiatheria species as the outgroup of Euarchontoglires it was suggested that the common ancestor of primates and rodents gave birth after systemic progesterone withdrawal and the functional withdrawal mechanism evolved later in the primates (Nnamani et al., 2013). The disparate mechanisms of progesterone maintenance and withdrawal in the two clades are underpinned by differences in cellular and molecular regulation, which limits the usefulness of common laboratory rats and mice in modeling parturition in the human (Mitchell and Taggart, 2009).

The guinea pig is considered a rodent species based on molecular phylogenetic analysis (Sullivan and Swofford, 1997), but delivers its offspring without a precipitous decline in maternal progesterone levels (Challis et al., 1971). Pregnant guinea pigs show reduced uterine progesterone responsiveness at term referred to as functional progesterone withdrawal (Porter, 1970). These primate-like features of progesterone action suggest that guinea pigs can be useful as an experimental model for human parturition. The phylogenetic split of the Neotropical caviomorphs from the African phiomorphs within the rodent infraorder Hystricognathi is estimated to have occurred about 42 million years ago (Murphy et al., 2007; Upham and Patterson, 2015), which is much later than the separation of Euarchonta and Glires. Therefore it is reasonable to infer that functional progesterone withdrawal evolved independently in primates and guinea pigs (Nnamani et al., 2013). Functional progesterone withdrawal is thus an analogous trait in guinea pigs and humans brought about by convergent evolution. This indicates the analogous phenotype characteristics may have arisen from different mechanisms at the cellular and molecular level. For example, maternal plasma progesterone concentrations are maintained in the guinea pig by a pregnancy-specific progesterone binding protein, which greatly reduces the metabolic clearance rate of the hormone (Illingworth et al., 1970; Challis et al., 1971; Evans et al., 1981). No such protein has been reported in humans indicating that a different mechanism, such as the high volume of placental progesterone production (Smith et al., 2009) is responsible for the elevated plasma progesterone concentration in pregnant women. This suggests that while closely modeling functional progesterone withdrawal, the analogous phenotype characteristics in the guinea pig may have arisen from different mechanisms at the cellular and molecular levels. However, the marked similarities can still be exploited to examine shared aspects of the withdrawal mechanism utilizing the advantages of animal experimentation.

### Mechanisms of Progesterone Withdrawal in Guinea Pigs and Humans

The decrease of target tissue responsiveness to biologically active concentrations of progesterone is termed progesterone withdrawal at the functional level. The principal progesterone target tissues that undergo functional progesterone withdrawal in humans and guinea pigs are the myometrium, cervix and the decidua, which is the endometrium of pregnancy (Mesiano et al., 2002; Merlino et al., 2007). These tissues express the nuclear progesterone receptors A and B (PRA and PRB), which are the products of the PGR/NR3C3 gene and mediate the genomic actions of the hormone. In labor, PRA expression increases relative to PRB in the human myometrium (Mesiano et al., 2002; Merlino et al., 2007). PRA has been shown to transrepress PRB-induced transcription in a cell type and promoter specific fashion (Vegeto et al., 1993), which suggests that the elevated PRA/PRB ratio may result in a blunted myometrial response to progesterone in agreement with functional progesterone withdrawal. This possibility was verified by Tan et al. (2012) using pregnant human myometrial cell lines engineered to be either PRB- or PRA-dominant. In PRB-dominant cells, progesterone upregulated the anti-inflammatory factor IκBα and suppressed numerous labor-promoting proinflammatory genes. In PRA-dominant cells, PRA repressed PRB-mediated actions and stimulated proinflammatory gene expression. Remarkably, inflammatory stimuli such as lipopolysaccharide and interleukin 1β increased PRA protein abundance and transrepression activity suggesting that inflammation may reduce myometrial progesterone responsiveness (Peters et al., 2017). Furthermore, Nadeem et al. have reported in a recent study (Nadeem et al., 2016) that progesterone level decreases in human myometrial cell nuclei in labor despite the persistently high circulating hormone concentration. The decrease is concomitant with the elevated myometrial expression of the progesterone metabolizing enzyme, 20α-HSD/AKR1C1. Nadeem and co-workers have also shown that in pregnant myometrial cells unliganded PRA and liganded PRB distribute predominantly to the nucleus, while liganded PRA and unliganded PRB reside mostly in the cytoplasm. Thus, local reduction of progesterone concentration may further increase the (unliganded) PRA to (liganded) PRB ratio in myometrial cell nuclei. This may bolster the activity of PRA to repress the anti-inflammatory action of PRB and to induce labor-promoting genes. Support for this possibility is provided by the observation that unliganded PRA stimulates the transcription of the key contraction-associated gene Cx45 in a transfected myometrial cell line (Nadeem et al., 2016).

Progesterone action in the other target tissues of pregnancy has not been investigated as thoroughly as in the myometrium. Available data strongly suggest, however, that in the progesterone responsive cell types of the cervix and the decidua, both PRA and PRB levels decrease in labor (Stjernholm-Vladic et al., 2004; Goldman et al., 2005; Lockwood et al., 2010). Overall decrease of progesterone receptor expression may decrease progesterone responsiveness and lead to functional progesterone withdrawal even without a detectable shift toward increased PRA/PRB ratio. Progesterone is metabolized in the cervix and in the decidua and enhanced metabolic inactivation has been proposed to occur in the cervix reinforcing the receptor-mediated functional progesterone withdrawal at labor (Andersson et al., 2008).

In the guinea pig uterus, progesterone receptor levels decline during the last third of gestation in normal and growth restricted pregnancies with no change in the PRA/PRB isoform ratio (Palliser et al., 2010; Welsh et al., 2014). A similar decline of total PR mRNA and protein expression has been reported in the guinea pig cervix between mid-gestation and late pregnancy (Rodriguez et al., 2003; Nnamani et al., 2013). Thus, the mechanism of functional progesterone withdrawal appears similar in the guinea pig and human gestational tissues with the exception of the myometrium, where progesterone receptor regulation in women is isoform specific. Furthermore, available evidence does not support the involvement of increased target tissue metabolism contributing to progesterone withdrawal in guinea pig gestational tissues at term (Wagner et al., 2017) contrary to humans (Nadeem et al., 2016). This may not be surprising, since a major proportion (≥90%) of circulating progesterone is bound to a pregnancy-specific binding protein in the guinea pig plasma as mentioned before resulting in low tissue progesterone levels in the uterus (Csapo et al., 1981).

Progesterone withdrawal causes gene expression changes in the gestational tissues that foster transition from a pregnancy-protective to a labor-promoting phenotype. Genome wide microarray analysis of the human myometrial cell transcriptome after manipulation of progesterone receptor levels in culture revealed that PRA and PRB influence distinct, but partially overlapping sets of genes (Tan et al., 2012). Several, but not all, PRB-induced genes are trans-repressed by PRA. Association with gene ontology (GO) term processes segregated the actions of PRA and PRB with PRA linked to proinflammatory and PRB to anti-inflammatory activities. One notable PRB target in myometrial cells is the repressive transcription factor ZEB1, which controls the expression of contraction-associated genes with the involvement of microRNAs. This pathway has been characterized in detail in mice (Renthal et al., 2010; Williams et al., 2012) but not in an animal model undergoing functional progesterone withdrawal. It is highly likely, however, that the two PR isoforms function as genuine transcription factors controlling distinct sets of genes. Unliganded PRA may also be active in the nuclei of myometrial cells, although this possibility has not been confirmed to date. Overall, functional progesterone withdrawal in women can be described more precisely as a transition from PRB-dominant to PRA-dominant gene regulation augmented by local progesterone metabolism and proinflammatory stimulation. The mechanism(s) driving this transition are still unclear particularly under non-infectious conditions such as normal pregnancy and idiopathic preterm labor. A caveat is that the model is based on data obtained by *in vitro* experimentation mostly using immortalized and gene-manipulated myometrial cell lines. Analysis of human uterine biopsies may provide suggestive *in vivo* evidence, but experimental variation of gestational length and targeted interventions in whole animals are crucial to establish the sequence of events and cause-effect relationships leading to diminished progesterone responsiveness at birth. These experiments require animal models with proven relevance to human parturition. Furthermore, the alternative functional progesterone withdrawal mechanism involving the decrease of both PR isoforms should be characterized in similar detail as the isoform switch in myometrial cells. This includes exploring intracellular PR distribution and isoform-specific gene regulation on a genome wide scale. The guinea pig appears the best non-primate candidate for obtaining these data by experimental manipulation of gestational length, hormonal levels, inflammatory load, oxidative stress, growth restriction and other adverse circumstances.

### Control of Labor by Prostaglandins

The eicosanoids prostaglandin E_2_ (PGE_2_) and prostaglandin F_2α_ (PGF_2α_) induce birth in humans and guinea pigs as observed in all mammalian species examined to date. Prostaglandin E_2_ and F_2α_ are produced in the gestational tissues, and their local actions include stimulating myometrial contractions, cervical remodeling and membrane rupture. Intrauterine prostaglandin levels are determined by the relative rates of biosynthesis and metabolism, and the balance of these processes results in low prostaglandin concentrations that are compatible with pregnancy maintenance. The balance changes at term resulting in elevated prostaglandin levels, which increase uterine contractility, soften the cervix and weaken the membranes. Importantly, in the guinea pig relaxation of the pubic symphysis is critical for a successful delivery. Rising PGE_2_ levels in this tissue contribute to the inflammatory changes that relax the pubic symphysis before labor. These observations suggest that PGE_2_ acts through mechanisms that are similar to those seen during human cervical dilatation (Rodriguez et al., 2003). Prostaglandin actions are pivotal for parturition to occur in both species, and understanding the mechanisms that control prostaglandin levels in the pregnant uterus is critical for understanding the regulation of the birth process (Mitchell et al., 1995; Zakar and Hertelendy, 2001).

**FIGURE 2:**
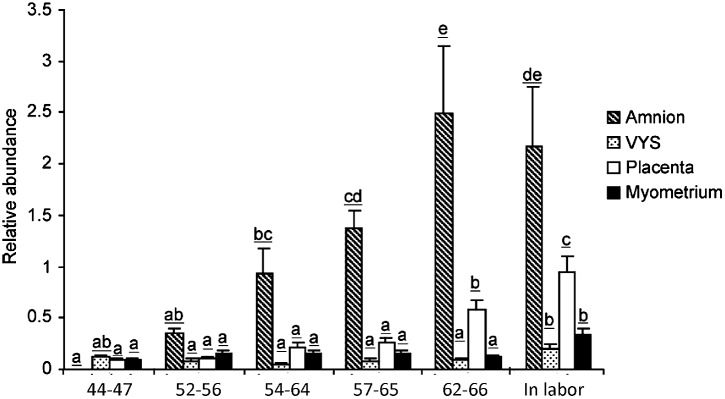
PTGS1 protein expression in guinea pig amnion, visceral yolk sac (VYS), placenta and myometrium during late pregnancy. Gestational age in days are indicated below the bars. The 57–65 days and 62–66 days groups correspond to the 1st and 5th days after pubic symphysis separation, respectively. The In labor group was collected after the birth of the first pup. Letters on top of the bars denote significance levels comparing gestational age groups for each tissue (*p* < 0.05, ANOVA with multiple comparison adjustment, *N* = 6 animals per group). Reproduced with permission from Welsh et al. (2005).

In the pregnant human uterus, a major source of labor-promoting PGE_2_ is the amniotic membrane. The capacity of the amnion to produce PGE_2_ increases sharply at term followed by a further rise during labor and also in preterm labor. The increase of synthetic capacity is due to the induction of PTGS2 (prostaglandin endoperoxide synthase-2), which is the enzyme catalyzing the irreversible committing step of prostaglandin biosynthesis. The other PTGS isoenzyme, PTGS1, remains unchanged. The biosynthetic precursor, arachidonic acid, is released from phospholipids by abundantly expressed phospholipases. Notably, the amnion is devoid of prostaglandin metabolic activity and functions as a prostaglandin producing tissue (Zakar and Hertelendy, 2001). Guinea pigs display remarkable similarities to the human. The amnion is a dominant source of intrauterine PGE_2_ in this species as well, and prostaglandin production by the guinea pig amnion increases markedly during the last third of gestation and with labor (Schellenberg and Kirkby, 1997). However, the PTGS isoform responsible for the rise of prostaglandin synthetic capacity is PTGS1 in the guinea pig (Welsh et al., 2005), which is the paralog of PTGS2 induced in women (Figure 2). PTGS1 is also upregulated in a growth restriction model causing preterm birth (Palliser et al., 2014). Furthermore, prostaglandin metabolism is very low in the guinea pig amnion, making the tissue an essential prostaglandin producer analogous to women. The incidence of preterm birth is higher in human pregnancies with a growth restricted fetus. Experimentally induced fetal growth restriction is also associated with reduced gestational length in guinea pigs, which suggests the guinea pig may be useful to uncover the pathways that trigger preterm labor in the presence of fetal compromise. Studies in the guinea pig have already shown that growth restriction induces an increase in uterine PTGS1 expression prior to delivery (Palliser et al., 2014).

The principal site of prostaglandin metabolism in the pregnant human uterus is the chorion. The chorion laeve is the membrane juxtaposed with the amnion and in contact with the decidua forming the fetal–maternal interface. Trophoblast cells in the chorion laeve express high levels of the prostaglandin metabolizing enzyme 15-hydroxy-prostaglandin dehydrogenase [NAD(++)], or HPGD/PGDH. The chorion laeve functions as a barrier to prevent the amnion-derived prostaglandins to reach other uterine tissues in active form. At labor, chorionic PGDH activity decreases allowing prostaglandins from the amnion to reach the myometrium without metabolic inactivation (Van Meir et al., 1997). Convergent evolution appears to result in a distinct, but analogous setting in the guinea pig. The chorion laeve deteriorates in guinea pigs at an early stage of development, and the fetal–maternal interface is formed between the endoderm-derived visceral yolk sac and the endometrium. The amnion is positioned side-by-side with the yolk sac membrane (Figure 1), which is highly vascularized, unlike the chorion laeve in humans. Prostaglandin metabolic activity is high in the guinea pig yolk sac due to abundantly expressed HPGD/PGDH (Welsh et al., 2012). Thus, the protective barrier function has been adopted by the visceral yolk sac membrane in guinea pigs by expressing the orthologous prostaglandin metabolic enzyme. HPGD expression decreases in the guinea pig yolk sac membrane during late gestation and in a growth restriction model of preterm birth (Welsh et al., 2012; Palliser et al., 2014), which may allow labor-inducing prostaglandins from the amnion to reach the myometrium similarly to what happens in the human.

### Involvement of Prostaglandins in Functional Progesterone Withdrawal

Clinical observations and *in vitro* experiments suggested that prostaglandins might influence PR expression and potentially contribute to functional progesterone withdrawal. In an immortalized human myometrial cell line, PGE_2_ and PGF_2α_ increased the PRA/PRB mRNA ratio concentration dependently through a protein kinase C-pathway (Madsen et al., 2004). Fibroblasts cultured from (non-pregnant) cervical tissue responded to interleukin-1β with transiently reduced PRA and PRB protein expression and increased PRA/PRB by ratio when the expression rebounded posttreatment. The transient decrease of PR levels, but not the subsequent rebound with increased PRA/PRB ratio, was blocked by the prostaglandin synthesis inhibitor indomethacin indicating the involvement of prostaglandins in the proinflammatory cytokine-induced drop in PR levels (Pierce et al., 2018). In the clinical setting, successful priming of the cervix by local administration of PGE_2_ in postterm pregnancies was associated with reduced (total) PR mRNA and protein levels in cervical biopsies (Vladic-Stjernholm et al., 2009). We have examined the involvement of prostaglandins in PR expression control experimentally in pregnant guinea pigs undergoing functional progesterone withdrawal (Welsh et al., 2014). Administration of the PGE_2_ analog sulprostone, a powerful abortifacient in this animal, induced preterm birth and significant reduction of PR mRNA and PRA protein levels, but not PRB protein levels, in uterine (myo-endometrium) tissue. Moreover, piroxicam, a prostaglandin synthesis inhibitor drug, delayed delivery and increased PRA protein abundance in the myo-endometrium compared to vehicle treated animals (Welsh et al., 2005, 2014). The use of the pregnant guinea pig model in this case provided strong *in vivo* evidence, not obtainable with humans, of the involvement of prostaglandins in PR-mediated functional progesterone withdrawal in the myometrium. The PR isoform changes varied between the humans and guinea pigs, but the overall effect of prostaglandins was consistent with decreasing progesterone responsiveness.

### The Roles of Relaxin at Birth

The involvement of the peptide hormone relaxin in the birth process was first discovered in guinea pigs (Dschietzig et al., 2006, for review). Relaxin was found an effective relaxant of the uterine muscle (Porter, 1971) and a powerful stimulant of pubic symphysis separation before labor. Later studies did not confirm a significant myometrial relaxant action in women (MacLennan et al., 1995). Elevated circulating maternal relaxin levels are associated with preterm birth (Weiss et al., 1993), however, which may be due to a role in cervical softening and fetal membrane remodeling caused by the proinflammatory actions of the hormone (Bryant-Greenwood et al., 2005). Relaxins have pleiotropic actions throughout the body including the brain (Baccari and Calamai, 2004; Dschietzig et al., 2006). Their role in perinatal brain development remains to be explored, and guinea pigs may prove useful for such studies.

## Modeling Preterm Labor in the Guinea Pig Following Pregnancy Compromise

### Growth Restriction Increases Vulnerability to Preterm Labor

The patterns of endocrine changes occurring in gestation in rats, mice and sheep differ markedly from those preceding labor in human and guinea pig pregnancy. The guinea pig provides a suitable model for investigating the role of *in utero* compromises in adverse pregnancy outcomes due to smaller litter sizes and a neurodevelopmental profile with considerable *in utero* brain development, which are features close to human pregnancy (Dobbing and Sands, 1979; Mitchell and Taggart, 2009). Placental insufficiency resulting in intrauterine growth restriction (IUGR) is a condition where the fetus is unable to achieve its genetically determined growth trajectory. IUGR is not only a leading cause of infant morbidity but is associated with a 2–3-fold increased risk of spontaneous preterm birth (Bukowski et al., 2001; Hershkovitz et al., 2001; Lackman et al., 2001). IUGR has been described as a late manifestation of early abnormal placental development. Notably, women with an episode of preterm uterine contractions who subsequently deliver at term are more likely to give birth to an IUGR infant (Espinoza et al., 2007). This suggests that compromises leading to IUGR increase the sensitivity of the uterus to stimulation, which may increase the risk of preterm labor. Previous studies suggest this vulnerability results from a premature, non-infectious upregulation of inflammatory and oxidative pathways in uterine tissues (Bisits et al., 2005; Moutsopoulos and Madianos, 2006; Romero et al., 2007; Lee et al., 2008). This shifts the balance of prostaglandin synthesis and metabolism toward production after the onset of growth restriction and well before labor onset (Palliser et al., 2014). We have established a surgically induced IUGR model in the guinea pig and observed increased incidence of spontaneous preterm birth in the growth restricted pregnancies (Palliser et al., 2014). This finding indicates that the timing of parturition in the guinea pig, like in the human, can be affected by IUGR, which supports the concept that there is early release of labor-inducing inflammatory mediators such as prostaglandins when fetal growth is chronically compromised.

### Role of Growth Restriction-Induced Inflammation in Preterm Labor

Intrauterine inflammation has a key role in the mechanism of labor at term, and early upregulation can lead to preterm uterine activation (Romero et al., 2007; Catalano et al., 2010). Labor onset is associated with the migration of leukocytes to the uterus, the release of proinflammatory cytokines and production of other inflammatory labor associated regulators including the key uterotonins PGF_2α_ and PGE_2_. The activation of this pathway culminates in inflammatory-mediated softening and dilatation of the cervix, weakening and rupture of the membranes and myometrial contractions (Illingworth et al., 1974; Csapo et al., 1981; Hirst et al., 1995; Thomson et al., 1999; Young et al., 2002; Palliser et al., 2004; Welsh et al., 2005). Previous studies have found that human IUGR pregnancies are characterized by an upregulation of proinflammatory cytokines including TNFα, interleukin (IL)-1β, IL-6, IL-8 and the down regulation of anti-inflammatory cytokine IL-10 (Heyborne et al., 1992; Hahn-Zoric et al., 2002; Bartha et al., 2003; Amu et al., 2006; Street et al., 2006; Sitras et al., 2009). Growth restriction has also been linked to the induction of the inflammatory cascade in the guinea pig uterus. ERK (extracellular signal-regulated protein kinase)-1/2 is activated in response to proinflammatory cytokines and oxidative stress mediators, which would turn on the intracellular signalling cascade responsible for nuclear factor kappa B (NFκB) activation reinforcing proinflammatory gene upregulation and promoting labor (Li et al., 2004; Otun et al., 2005; Sooranna et al., 2005). In agreement with this, NFκB DNA binding activity increases in human gestational tissues at both term and preterm labor (Belt et al., 1999; Allport et al., 2001; Yan et al., 2002a) and activation is functionally linked to the expression of key labor mediators including prostaglandin synthetic enzymes phospholipase A_2_ (PLA_2_), PTGS, TNFα, IL-1β, IL-6, and IL-8 (Baldwin, 1996; Belt et al., 1999; Allport et al., 2001; Yan et al., 2002b). The increase in these mediators, particularly prostaglandins, in guinea pig IUGR pregnancies would indicate the upregulation of these NFκB driven inflammatory pathways, which may lower the threshold to trigger the onset of labor.

**FIGURE 3:**
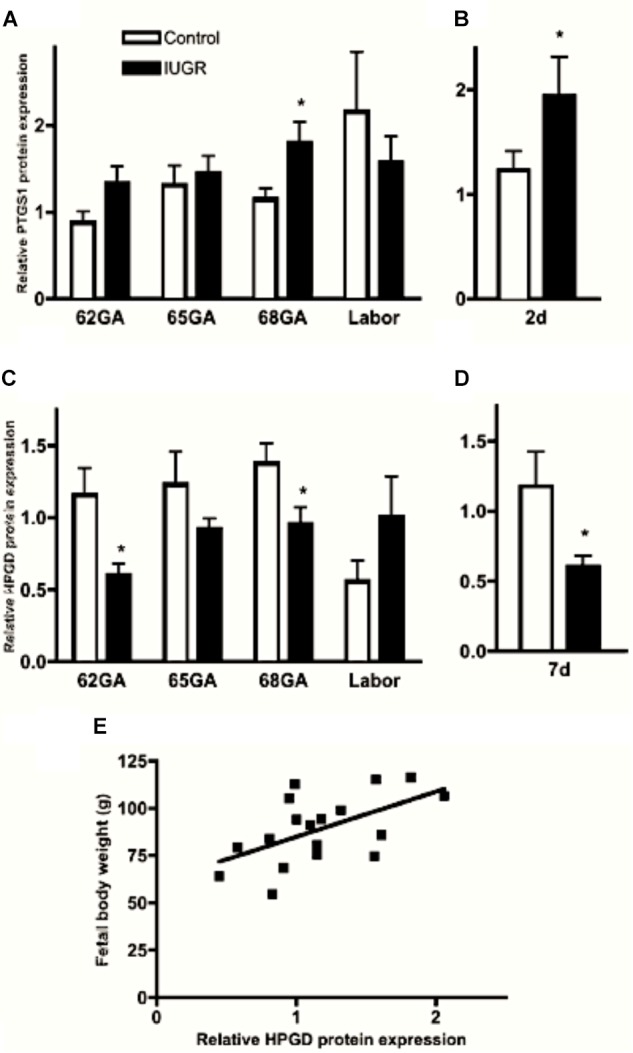
Myometrial PTGS1 **(A,B)** and visceral yolk sac membrane HPGD **(C,D)** protein expression in control (open bars) and IUGR (closed bars) guinea pig pregnancies over late gestation and 2 **(B)** and 7 **(D)** days prior to expected delivery. ^∗^*P* < 0.05 IUGR vs. control. Significant positive correlation between fetal body weight and visceral yolk sac membrane HPGD levels at GA68 (**E**; *r* = 0.56, *P* = 0.015). Adapted with permission from Palliser et al. (2014).

Proinflammatory cytokines stimulate uterine PTGS expression and inhibit prostaglandin metabolism by 15-hydroxyprostaglandin dehydrogenase (HPGD) thereby promoting a high prostaglandin environment (Brown et al., 1998; Mitchell et al., 2000). Myometrial PTGS expression is prematurely elevated in IUGR-associated pregnancies in the guinea pig and IUGR pregnancies are associated with significant down regulation of HPGD expression in the visceral yolk sac membrane (Figure 1), which assumes the role of the chorion laeve in many other mammals (Figure 3), (Kaufmann and Davidoff, 1977; Palliser et al., 2014). We have proposed that this changes the balance of prostaglandin synthesis and metabolism in late gestation, producing an elevated prostaglandin environment leaving these pregnancies vulnerable to further stimulation that may trigger preterm labor. Indeed we found there was a significant association between reduced fetal body weight and reduced capacity of the visceral yolk sac membrane to metabolize prostaglandins (Figure 3E; Palliser et al., 2014). Interestingly, this premature decline in HPGD expression was not merely a reflection of the shorter gestational length of these pregnancies. There was a marked suppression of HPGD more than a week prior to term in IUGR pregnancies (Figure 3D; Palliser et al., 2014) suggesting the presence of an inflammatory environment in these compromised pregnancies well in advance of labor. This further indicates that compromised pregnancies result in increased vulnerability to triggers of labor before term.

### Effects of IUGR-Induced Inflammation on Progesterone Receptor Signaling

**FIGURE 4:**
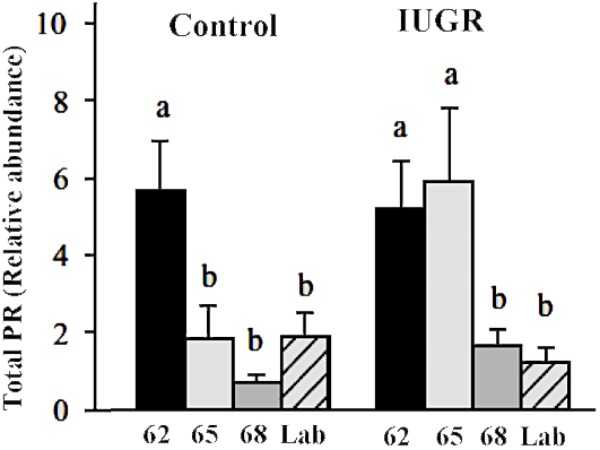
Myometrial PR expression at 62, 65, 68 days of gestation and at labor in the guinea pig. Different letters indicate significant difference at *P* < 0.05. Adapted with permission from Palliser et al. (2010).

There is a significant difference in progesterone receptor expression between myometrial samples from IUGR associated pregnancies and those from control pregnancies (Figure 4; Palliser et al., 2010). Myometria from growth restricted pregnancies showed down regulation of PRA and PRB later during pregnancy compared to control pregnant guinea pigs (>65 days of gestation compared to 62 days in controls). The prolonged PR expression in the setting of IUGR promotes myometrial quiescence in the face of rising preterm uterine activation involving PTGS and HPGD. As delivery occurred at a significantly earlier gestational age in IUGR, the preservation of PRs expression and action was not sufficient to prolong IUGR associated pregnancies to term. This notion was supported by the observation that IUGR pregnancies delivered 4 days after the decrease of myometrial PRs (65–69 days) whilst control (sham-operated) pregnancies delivered 9 days after the decrease in PRs expression (62–71 days). Furthermore, the size of the guinea pig makes implantation of transponders highly feasible and allows recording of uterine activity from mid gestation to labor. IUGR-associated pregnancies showed a more rapid increase in contractile activity and a shorter period of time from a relatively quiescent state to the time of delivery (Palliser et al., 2014) than normal pregnancies. These observations further support the contention that IUGR pregnancies are more responsive to uterine stimulation even in the presence of sustained PR expression.

## Outcomes Following Preterm Birth

### Preterm Birth Leads to Neurodevelopmental Deficits

There are very limited options for preventing spontaneous preterm birth. The majority (∼84%) of human preterm birth cases fall into the moderate to late preterm range, which is associated with relatively low mortality in developed countries (Blencowe et al., 2012). The adverse postnatal outcomes for this group, however, received little attention. This age range can be readily studied in guinea pigs as pups can be delivered at 7–9 days preterm but have neurological deficits similar to those seen in moderate to late human preterm neonates (Shaw et al., 2016). There is substantial clinical evidence that moderate to late preterm birth is associated with cognitive and behavioral problems in later life (Kerr-Wilson et al., 2012; Kugelman and Colin, 2013; Spittle et al., 2016a). There is also growing evidence pointing to a disruption of dendritic development, neurite pruning and myelination at late gestation forming a link between prematurity and adverse postnatal outcomes such as behavioral disorders in childhood and adolescence (Back and Miller, 2014; Kelly et al., 2016; van Tilborg et al., 2016).

The 69–71 day relatively long gestation of the guinea pig makes this species particularly useful for modeling the effects of moderate to late preterm birth on the offspring. Pups can be delivered at 62–63 days of pregnancy and survive although need considerable postnatal care (Dyson et al., 2012; Morrison et al., 2018). Delivery at this time results in the loss of the *in utero* environment for a substantial period compared to delivery at term, which may result in a marked adverse neurological outcome. The interval from 62 days of gestation until term is a time of rapid maturation of OPCs (oligodendrocyte precursor cells) to myelin synthesizing oligodendrocytes and therefore delivery at this age results in a deficit in the number of mature oligodendrocytes (Tolcos et al., 2011). This leads to reduced myelination with deficits detectable at term equivalent age (Shaw et al., 2016). Recent studies in the guinea pig have shown that the reduced myelination in preterm pups persists until at least 1 month of age, the equivalent of human early childhood (Palliser et al., 2015; Shaw et al., 2016). These observations suggest that premature transition to *ex utero* life delays myelination for a considerable time after birth due to losing *in utero* exposure to neurotropic and gliotrophic factors critical for normal brain development. In addition, the guinea pig studies have shown that preterm birth-induced deficits are more marked in the male offspring, which is consistent with the greater incidence of adverse outcomes in preterm delivered male human infants (Shaw et al., 2016).

### Effects of IUGR on Neurodevelopment

IUGR is associated with prematurity-related neurodevelopmental impairments (Kugelman and Colin, 2013; Spittle et al., 2016a,b). In addition, there is an independent association of IUGR with poor neonatal outcome and delayed neurodevelopment. There are parallels and links between the inflammatory and oxidative processes that lead to vulnerability to preterm labor in IUGR pregnancies and those that contribute to the developmental delays and injury in the IUGR fetal brain. For example, proinflammatory cytokines are able to cross the immature blood brain barrier and activate oxidative processes. These include stimulation of NO, free radical production and inflammatory processes including CNS cytokine production, excitotoxicity, breakdown of the blood brain barrier (Chao et al., 1996) and OPCs impairment (Pang et al., 2000). Using guinea pigs, we have shown decreased myelination following induced -IUGR, which was not the consequence of preterm birth (Kelleher et al., 2011).

### Sex Dependence in Behavioral Disorders Following Preterm Birth

Moderate to late preterm birth is associated with behavioral disorders and academic disadvantage. These include increased incidence of ADHD (attention deficit hyperactivity disorder) and anxiety in children delivered preterm, which often appear in childhood and adolescence. Studies in preterm guinea pigs, delivered at 62 days, have demonstrated marked changes in behavioral patterns with males showing hyperactivity and females showing increased anxiety (Shaw et al., 2016). These disorders were associated with reduced myelination and reactive astrocyte coverage in the hippocampus and subcortical white matter in preterm males, which suggests that deficient maturation of the oligodendrocyte linage contributes to these neurobehavioral pathologies. There appears to be a catch up in myelination after term equivalent age; however, increased levels of behavioral problems remain (Shaw et al., 2016). The mechanisms contributing to the gender-associated adverse behavioral patterns before sexual maturity remain unclear. Possible causative processes include the premature loss of intra-uterine and placental factors that support late gestation neurodevelopment, which may vary depending on fetal sex. Together these findings illustrate the utility of the guinea pig model to explore approaches to improve long-term neurodevelopmental outcomes after preterm birth.

### Preterm Birth Leads to Neurosteroid Deficiencies

Progesterone, in addition to maintaining pregnancy, has a key role in CNS (central nervous system) activity and development by direct action and by providing precursors for neurotrophic steroids (Hirst et al., 2014). Placental progesterone synthesis leads to concentrations of the protective and trophic neurosteroid, allopregnanolone, in the fetal brain that are markedly higher than at any other time during life (Hirst et al., 2014). Allopregnanolone does not interact with progesterone receptors but binds as a potent agonist to a steroid binding site on the GABA_A_ receptor, which promotes the opening of the associated Cl^-^ ion channel. GABA_A_ receptor stimulation in brain tissue from preterm human neonates and in the guinea pig fetus has shown the suppression of CNS activity by ∼0.6 of gestation (or 24–26 weeks of human pregnancy (Kahle and Staley, 2008; Ben-Ari et al., 2012). This supports the contention that high levels of allopregnanolone during late gestation maintain tonic suppression of excitability that protects the brain from excitotoxic damage (Hirst et al., 2014; Schumacher et al., 2014). In contrast, stimulation of this receptor earlier in gestation may have neurotrophic action (Zonouzi et al., 2015). These observations suggest that the developmental transition of GABA_A_ receptor function during gestation controls the action of progesterone and its neuroactive derivatives in the fetal brain in both species.

In the guinea pig, as in primates, birth is followed by a precipitous fall in brain neurosteroids as a consequence of the loss of the placental supply of neurosteroids and their precursor, progesterone (Kelleher et al., 2013; Hirst et al., 2014). Notably, progesterone administration to pregnant guinea pigs raised maternal levels, but had no effect on progesterone or allopregnanolone levels in the fetal plasma or brain (Kelleher et al., 2013). In contrast, progesterone administration to preterm neonates markedly elevated the level of the potent neurosteroid, allopregnanolone, in the brain (Palliser et al., 2015). These findings indicate the important role of the placenta in regulating neurosteroid and neurosteroid precursors reaching the brain during gestation. Moreover, they may have clinical implications as high doses of progesterone often administered as a vaginal pessary to reduce the risk of preterm birth. While there is little information to indicate if the levels of progesterone metabolites in the human fetus are raised with progesterone therapy in mid gestation, findings in the guinea pig suggest the placenta controls passage and levels in the fetus such that these treatments are unlikely to affect neurosteroid levels in the fetal brain (Kelleher et al., 2013).

Based on the observations outlined above it is reasonable to conjecture that progesterone treatment of preterm neonates may be beneficial for neuroprotection. Indeed, progesterone treatment was shown to raise progesterone and allopregnanolone levels in the circulation and brain of preterm guinea pig neonates (Kelleher et al., 2013). However, the expected protective actions were blunted by cortisol overproduction driven by the precursor, progesterone, particularly in males (Palliser et al., 2015). It appears the dose of progesterone administered to neonates is critical in the absence of the regulatory role of the placenta (Kelleher et al., 2013). Work with the guinea pig suggests care is needed with neonatal steroid replacement treatments to achieve appropriate neuroprotective effects in the preterm neonatal brain.

## Concluding Remarks

Similarities between humans and guinea pigs in placental anatomy, gestational timing and the endocrine/paracrine regulation of pregnancy have long been recognized leading to the suggestion that the guinea pig is the most informative non-primate model of human pregnancy and parturition (Young, 2001; Mitchell and Taggart, 2009). The key parallels include birth at high maternal progesterone levels, functional progesterone withdrawal mediated by altered progesterone receptor expression at labor, the involvement of prostaglandins in progesterone receptor regulation and anatomical separation of prostaglandin synthesis and metabolism in the fetal membranes. These similarities appear to have evolved independently in humans and guinea pigs and represent analogous traits, rather than homologies derived from a common ancestor. Convergent evolution may explain intriguing differences such as the disparate ways of maintaining circulating progesterone levels in the mother (high synthesis vs. low clearance rates), upregulation of alternative paralogs of PTGS (PTGS2 vs. PTGS1) in the amnion before birth, different PR isoform expression profiles in the myometrium at functional progesterone withdrawal and the presence of ontogenetically distinct membranes (chorion leave vs. visceral yolk sac) juxtaposed to the amnion and expressing the orthologous prostaglandin metabolizing enzyme, HPGD/PGDH. Nonetheless, dissimilarities did not weaken the usefulness of the guinea pig as an experimental model of human pregnancy. This is illustrated best by the use of guinea pigs for the development of safe non-surgical abortion methods employing the combination of anti-progesterone and prostaglandins (Elger et al., 1987). This procedure is now in regular clinical use worldwide (Raymond et al., 2013). Furthermore, guinea pig models of the major pregnancy pathologies including preterm birth (Elger et al., 1987; Bytautiene et al., 2004; Mitchell and Taggart, 2009), preeclampsia (Seidl et al., 1979; Golden et al., 1980) and even Zika virus infection (Kumar et al., 2017) are available. Surgical manipulation of uterine/placental blood supply allows reproducible modeling of IUGR (Turner and Trudinger, 2009; Palliser et al., 2010). These disease models have excellent translational potential. For example, the IUGR model revealed increased incidence of preterm birth, a shift toward prostaglandin synthesis in the gestational tissues and advanced transition from a quiescent to a labor-associated pattern of uterine EMG activity after growth restriction. Behavioral disorders were detected in premature offspring particularly in males, which was associated with persistently impaired brain structure. Alterations in brain development were shown in IUGR, which was independent of preterm birth.

The guinea pig preterm parturition model was particularly useful for exploring conditions of neurosteroid deficiency and the possibility of neurosteroid replacement therapy in the preterm newborns. The precocial developmental state of guinea pig newborns is similar to primates with more of brain development occurring *in utero* and stimulated by the nurturing *in utero* steroid environment. The gestational levels of progesterone support the maintenance of high levels of neurosteroids, which are essential for normal brain development. Brain maturation in the guinea pig includes the change of GABAergic stimulation to inhibition at mid-to-late pregnancy, which also occurs in the human. This makes the guinea pig a superior model for examining the action of GABA_A_ receptor-neurosteroid interaction and the for studying neuroprotective processes. The comparatively advanced neurodevelopment of the pups makes the guinea pig particularly useful for examining the impact of perinatal compromises on long term neurobehavioral outcomes. Preventative therapies could also be designed with strong translational potential to human pregnancy and newborn outcome.

## Author Contributions

Manuscript concept and design were by JH and TZ. Contribution by DW was intellectual. Manuscript draft was prepared by JH, HP, JS, GC, and TZ. Final editing was done by JH and TZ.

## Conflict of Interest Statement

The authors declare that the research was conducted in the absence of any commercial or financial relationships that could be construed as a potential conflict of interest.
